# Molecular epidemiology of *Clostridioides difficile* in companion animals: Genetic overlap with human strains and public health concerns

**DOI:** 10.3389/fpubh.2022.1070258

**Published:** 2023-01-06

**Authors:** Frederico Alves, Rita Castro, Miguel Pinto, Alexandra Nunes, Constança Pomba, Manuela Oliveira, Leonor Silveira, João Paulo Gomes, Mónica Oleastro

**Affiliations:** ^1^National Reference Laboratory of Gastrointestinal Infections, Department of Infectious Diseases, National Institute of Health Doutor Ricardo Jorge (INSA), Lisbon, Portugal; ^2^Genomics and Bioinformatics Unit, Department of Infectious Diseases, National Institute of Health Doutor Ricardo Jorge (INSA), Lisbon, Portugal; ^3^Faculty of Veterinary Medicine, Lusófona University, Lisbon, Portugal; ^4^Genevet–Veterinary Molecular Diagnostic Laboratory, Carnaxide, Portugal; ^5^CIISA–Centre for Interdisciplinary Research in Animal Health, Faculty of Veterinary Medicine, University of Lisbon, Lisbon, Portugal

**Keywords:** *Clostridioides difficile*, companion animals, one health, whole genome sequencing, SNP analysis, antimicrobial resistance, CDI trends

## Abstract

**Introduction:**

The changing epidemiology of *Clostridioides difficile* reflects a well-established and intricate community transmission network. With rising numbers of reported community-acquired infections, recent studies tried to identify the role played by non-human reservoirs in the pathogen's transmission chain. This study aimed at describing the *C. difficile* strains circulating in canine and feline populations, and to evaluate their genetic overlap with human strains to assess the possibility of interspecies transmission.

**Methods:**

Fecal samples from dogs (*n* = 335) and cats (*n* = 140) were collected from two populations (group A and group B) in Portugal. *C. difficile* isolates were characterized for toxigenic profile and PCR-ribotyping. The presence of genetic determinants of antimicrobial resistance was assessed in all phenotypically resistant isolates. To evaluate the genetic overlap between companion animals and human isolates from Portugal, RT106 (*n* = 42) and RT014/020 (*n* = 41) strains from both sources were subjected to whole genome sequencing and integrated with previously sequenced RT106 (*n* = 43) and RT014/020 (*n* = 142) genomes from different countries. The genetic overlap was assessed based on core-single nucleotide polymorphism (SNP) using a threshold of 2 SNP.

**Results:**

The overall positivity rate for *C. difficile* was 26% (76/292) in group A and 18.6% (34/183) in group B. Toxigenic strains accounted for 50% (38/76) and 52.9% (18/34) of animal carriage rates, respectively. The most prevalent ribotypes (RT) were the toxigenic RT106 and RT014/020, and the non-toxigenic RT010 and RT009. Antimicrobial resistance was found for clindamycin (27.9%), metronidazole (17.1%) and moxifloxacin (12.4%), associated with the presence of the *ermB* gene, the pCD-METRO plasmid and point mutations in the *gyrA* gene, respectively. Both RT106 and RT014/020 genetic analysis revealed several clusters integrating isolates from animal and human sources, supporting the possibility of clonal interspecies transmission or a shared environmental contamination source.

**Discussion:**

This study shows that companion animals may constitute a source of infection of toxigenic and antimicrobial resistant human associated *C. difficile* isolates. Additionally, it contributes with important data on the genetic proximity between *C. difficile* isolates from both sources, adding new information to guide future work on the role of animal reservoirs in the establishment of community associated transmission networks and alerting for potential public health risk.

## Introduction

Since its first discovery, *Clostridioides difficile* has been recognized as the number one cause of hospital acquired antibiotic associated diarrhea in humans, with severe infections developing into pseudomembranous colitis (1). Symptomatic infections usually arise from a disturbance of the gastrointestinal microbiota following antimicrobial administration and are due to the production of the bacterium's main virulence factors, toxins A and B (2, 3). Epidemiologic surveillance programs show that *C. difficile* infections (CDI) are trending from nosocomial toward a more community acquired infection (4).

With a growing awareness in regard to this reality and a clearer case definition, several studies have been reporting concerning rates of community-acquired *C. difficile* infection (CA-CDI) that can be as high as 41% (5). A rising number of studies report the isolation of *C. difficile* in food producing animal feces (6–8), abattoir samples (9), food products (10) and environmental samples (11), reinforcing the importance of understanding the potential role assumed by these reservoirs in CDI epidemiology. While much attention has been drawn to the possibility of this pathogen circulating in the human food chain, the relatively low prevalence rate found in retail food products in European countries (12) and the sparse contact the general population holds with food producing animals raises questions about the real impact this transmission route has on CA-CDI cases.

In more recent years, research focused on the role of companion animals as possible reservoirs for human CDI has been growing, with results suggesting that they may constitute a potential public health risk (13, 14). Most studies support the idea that dogs are mainly asymptomatic carriers with no significant clinical presentation arising from toxigenic *C. difficile* colonization. The prevalence varies between studies but values usually fall below the 20% mark (15–17), with higher prevalence rates being mostly reported by studies focused on neonatal populations (18). Studies with cats as the target population are sparse but they appear to follow the same trend as dogs, with a similar low prevalence of asymptomatic carriers being reported by the studies that consider both species (6, 17). The asymptomatic carrier status of most colonized companion animals may constitute an additional risk factor for *C. difficile* dissemination, as the absence of gastrointestinal signs decreases public perception of the pathogenic potential of the fecal material (19), thus enabling a silent spread.

The concerns raised regarding the possible role of companion animals in the community transmission network have been strengthened by studies showing some degree of genetic overlap between isolates from these animals and humans (14). While these results might be suggestive of a possible interspecies transmission, most studies are restricted to evaluating genetic proximity at ribotype (RT) level, which lacks the resolution power to assume a probable genetic link. A more in-depth genomic analysis is necessary to better understand how isolates from different species correlate before assuming any transmission route.

The social changes surrounding companion animals -human relations in modern society favored lifestyle and behavioral changes that led to their increased proximity, with the conditions needed for interspecies transmission to occur increasingly present in the animal owning households. It is thus essential that the One Health approach is broadened to include not only food producing animals and the environment, but also companion animals, especially when the concept is applied to community circulating pathogens like *C. difficile*.

Increasing scientific findings pointing to dogs and cats as possible reservoirs of toxigenic *C. difficile* strains are bringing companion animals to the center stage of the CA-CDI panorama. This study provides another contribution to this field, aiming at characterizing the circulating *C. difficile* strains present in companion animals' feces, as well as evaluating the genetic proximity between animal and human strains, in order to add new information on the epidemiological role of these animals in CA-CDI.

## Materials and methods

### Animal samples collection

The 475 fecal samples from dogs and cats included in this study were prospectively collected by means of convenience sampling and grouped considering the sampling context. One group of animals (group A, *n* = 292) was sampled by veterinary professionals at two veterinary hospitals in Portugal, located at the two biggest and most populated Portuguese urban centers, between July and August 2021. These animals were attending the veterinary hospital for different medical reasons that could or not be of gastrointestinal origin. Stool samples were obtained either by rectal swab or by digital rectal collection depending on animal size, and each sample was accompanied by a questionnaire briefly covering the recent clinical history (antibiotic administration and stool consistency), demographic data and environmental living conditions of each animal (in Supplementary material). In accordance with hospital practices, before sampling, owners signed an informed consent agreeing with the use of fecal samples for investigation purposes. The statistical analysis to assess the correlation between altered fecal consistency and the presence of *C. difficile* was performed using Fisher's exact test and a *p*-value of < 0.05 was considered significant. The other group of samples (group B, *n* = 183) was provided by a veterinary diagnostic laboratory which receives samples from several Portuguese veterinary hospitals and clinics. These samples were either collected by veterinary professionals or by animal owners following veterinary advice, between November 2020 and June 2021, and sent to the laboratory for further investigation. Group B samples belonged to animals with indication for a fecal exam due to gastrointestinal signs of disease following evaluation by the assisting veterinary surgeon.

### Isolation of *Clostridioides difficile*

Around 0.5 g of each stool sample was enriched in 5 ml of *C. difficile* enrichment broth (proteose peptone 40.0 g/L, disodium hydrogen phosphate 5.0 g/L, potassium dihydrogen phosphate 1.0 g/L, magnesium sulfate 0.1 g/L, sodium chloride 2.0 g/L, fructose 6.0 g/L, sodium taurocholate 1.0 g/L, D-cycloserine 0.25 g/L, cefoxitin 8.0 mg/L) for a week under anaerobic conditions, generated using the anaerobic cultivation system Anoxomat (Anoxomat, Mart), at 37°C, with atmosphere renewal every 48 h. For rectal swabs, these were directly inoculated in 5 mL of *C. difficile* enrichment broth. Following this step, all samples were subjected to ethanol shock (2.5 mL of the enrichment mixture in 2.5 mL of 96–100% ethanol for 1 h at room temperature) and centrifugation (3500 rpm for 10 min) before inoculating the resulting pellet onto ChromID^®^ *C. difficile* agar (bioMérieux, Marcy l'Etoile, France) for 48–72 h under anaerobic conditions at 37°C.

### Toxin profile, ribotyping and antimicrobial resistance

Each sample was assessed for the presence of *C. difficile* based on colony morphology. From each presumably positive sample, four colonies were picked and cultured onto Brain Heart infusion agar (Oxoid™, Madrid, Spain) under anaerobic conditions for 24 h at 37 °C. After species confirmation by MALDI-TOF (VITEK^®^ MS, bioMérieux), genomic DNA was extracted using the Isolate II Genomic DNA kit (Bioline, London, United Kingdom), according to manufacturer's instructions. Each isolate was characterized by multiplex PCR, targeting *gluD* and the *tcdA, tcdB, cdtA* and *cdtB* toxin genes, according to Persson et al. (20), and by PCR-ribotyping using Bidet primers (21) followed by capillary gel-based electrophoresis, according to Fawley et al. (22). The RT was determined using the Webribo database (https://webribo.ages.at/). For numeration purposes, in cases where the four colonies belonged to the same RT, only one isolate was considered. Antimicrobial susceptibility was performed by disc diffusion (Oxoid ™), using the discs and zone diameter breakpoints described by Erikstrup et al. (23): moxifloxacin (5 μg, ≥20 mm), vancomycin (5 μg, ≥19 mm), metronidazole (5 μg, ≥23 mm) and rifampicin (5 μg, ≥20 mm). For clindamycin the Etest^®^ strips (bioMerieux) were used and strains were categorized according to the Clinical & Laboratory Standards Institute breakpoint (≥8 mg/L) (24). Brucella blood agar supplemented with hemin and vitamin K1 (BD BBLTM, Heidelberg, Germany) was used; plates were incubated for 24 h under anaerobic conditions. The reference *C. difficile* strain ATCC 700057 was included for quality control.

### Whole genome sequencing and assembly

For the present study, 83 *C. difficile* isolates from Portugal, belonging to contemporary human CDI cases (*n* = 41), canines (*n* = 33) and felines (*n* = 9) from the present study, all belonging to the main toxinogenic types found, RT014/RT020 and RT106, were considered for deeper genetic analysis by whole genome sequencing (WGS). DNA was subjected to Nextera XT library preparation (Illumina, San Diego, CA, USA) prior to paired-end sequencing (2 × 250 bp or 2 × 150 bp) on either a MiSeq, NextSeq 550 or NextSeq 2000 instrument (Illumina), according to the manufacturer's instructions. For integration purposes, raw reads datasets for *C. difficile* RT014/RT020 and RT106 were retrieved from the European Nucleotide Archive (ENA), after query in the Enterobase platform (25, 26) and from previously published studies (14, 27). All genome sequences were assembled using the INNUca v4.2.0 pipeline (https://github.com/B-UMMI/INNUca), an integrative bioinformatics pipeline for read quality analysis and improvement and *de novo* genome assembly and polishing (28). Multi-Locus Sequence Typing (MLST) was performed using *mlst* v2.16.1 software (https://github.com/tseemann/mlst). Isolate metadata and genome statistics are detailed in Supplementary Tables S1,S2. Genome annotation was performed using Prokka v1.14.5-2 software (https://github.com/tseemann/prokka) (29).

### Comparative genomic analysis and phylogeny

In order to compare the genome background of *C. difficile* isolates collected from human infections and companion animals, RT014/R020 and RT106 quality-processed reads were mapped against reference genomes S-0352 (RT014) (Genbank Accession number CP076377.1) and DH/NAP11/106/ST-42 (CP022524.1), respectively, using Snippy v.4.5.1 (https://github.com/tseemann/snippy; –mincov 10, –minfrac 0.7, –mapqual 30, –basequal 20). Core-single nucleotide polymorphism (SNP) were extracted using Snippy's core module (*snippy-core*) ensuring that all genomes reached at least 70% of aligned bases with the reference (30) (which occurred for all sequenced samples). Core-single nucleotide variant falling within genetic islands [identified using IslandViewer 4 (31)] and potential phage regions [identified using PHASTER (32)] were excluded as these may bias the phylogeny (Supplementary Table S3). Minimum spanning trees (MST) were constructed using GrapeTree (33). Genetic clusters with potential epidemiological relevance, i.e., as potential short-term transmission, were defined at a SNP distance threshold of ≤ 2, as previously reported (30). Cluster metadata composition were retrieved using the ReporTree software (34) (https://github.com/insapathogenomics/ReporTree).

### Antimicrobial resistance genetic markers and mobile genetic elements

For all RT014/RT020 and RT106 isolates subjected to WGS, ABRicate v.1.0.0 (https://github.com/tseemann/abricate) was used to screen for *in silico* antimicrobial resistance (AMR) genetic determinants against ResFinder (35), NCBI AMRFinderPlus (36), ARG-ANNOT (37), CARD (38) and MEGARes (35) databases (last updated on August 3, 2022). For all isolates showing phenotypic resistance from other RTs, the following AMR genetic determinants were screened by PCR and/or Sanger sequencing: the *ermB* gene for clindamycin, ORFs 8 and 6 of the pCD-METRO plasmid for metronidazole, *rpoB* for rifampicin and *gyrA/gyrB* for fluoroquinolones, respectively, as previously described (39–41).

The existence of plasmids was assessed through PlasmidFinder 2.1 (42), whereas the search for prophages was also performed with PHASTER web server (32). All RT106 isolates were further screened by Snippy reference-based mapping for the presence of the known genomic islands (GIs): the RT106-characteristic GI1 (~46kb) (43) GI2 (~46kb) (44) and GI3 (~29kb) (43).

### Data availability

All reads generated for the present study were deposited in the ENA under the study accession number PRJEB49792 (individual run accession numbers are detailed in Supplementary Table S1).

## Results

### Population characteristics and *Clostridioides difficile* positivity rate

Regarding population characteristics of group A (veterinary hospitals samples), 80.5% (235/292) were canine and 19.5% (57/292) were feline samples; most of the animals, 49% (143/292), were 1–8 years old (Table 1). Considering clinical information, 20.6% (60/292) had diarrhea and 78.4% (229/292) were not showing gastrointestinal signs at the time of sampling; 41.8% (122/292) had no information concerning antibiotic administration, 3.1% (9/292) had a recent history of antibiotic administration (here considered as an antibiotic administration from 3 months to a week preceding the sample collection) and 55.1% (161/292) had not recently taken any antibiotic. In group A, the overall positivity rate for *C. difficile* was 26% (76/292), being slightly more frequently detected in dogs (27.2%; 64/235) than in cats (21.2%; 12/57). The positivity rate by age group did not show considerable differences. Regarding gastrointestinal signs, the positivity rate in diarrheic animals, 36.7% (22/60), was considerably higher than in animals with normal fecal consistency, 22.7% (52/229). Nevertheless, there was no statistically significant association between the isolation of toxigenic *C. difficile* strains and an altered fecal consistency (*p* = 0.4468). Also, there was no relevant difference between the isolation rate of *C. difficile* and here considered antibiotic administration. The positivity rate distribution by variable is summarized in the Table 1.

**Table 1:** Population characteristics and *Clostridioides difficile* positivity rate in groups A and B.

**Variable**	**Parameters**	***n* (%)**	**Positive *n* (%)**
**Group A (veterinary hospitals)**			
Species	Canine	235 (80.5%)	64 (27.2%)
	Feline	57 (19.5%)	12 (21.1%)
Sex	Male	152 (52.1%)	35 (23%)
	Female	140 (47.9%)	41 (29.3%)
Age	< 1	36 (12.3%)	8 (22.2%)
	1–8	143 (49%)	40 (28%)
	>8	113 (38.7%)	28 (24.8%)
Diarrhea	Yes	60 (20.6%)	22 (36.7%)
	No	229 (78.4%)	52 (22.7%)
	Unknown	3 (1%)	2 (66.7%)
ATB	Yes	9 (3.1%)	2 (22.2%)
	No	161 (55.1%)	47 (29.2%)
	Unknown	122 (41.8%)	27 (22.1%)
**Group B (veterinary diagnostic laboratory)**			
Species	Canine	100 (54.6%)	23 (23%)
	Feline	83 (45.4%)	11 (13.3%)
Sex	Male	85 (46.4%)	19 (22.4%)
	Female	78 (42.6%)	15 (19.2%)
	Unknown	20 (10.9%)	-
Age	< 1	54 (29.5%)	6 (11.1%)
	1–8	83 (45.4%)	20 (24.1%)
	>8	20 (10.9%)	3 (15%)
	Unknown	26 (14.2%)	4 (15.4%)

In group B (veterinary diagnostic laboratory), the species distribution was 54.6% (100/183) canine and 45.4% (83/183) feline; most animals, 45.4% (83/183) were 1–8 years old (Table 1). All animals in this group had a clinical history of chronic diarrhea or gastrointestinal disease. None of the animals had information regarding recent antibiotic administration. *C. difficile* overall positivity rate found in group B was 18.6% (34/183), with 23% (23/100) of the dogs included carrying *C. difficile* in their feces against 13.3% (11/83) of cats. There was a considerably higher rate in the 1-8 years old group, 24.1% (20/83), than in the remaining age groups. Data regarding the positivity rate by variable is summarized in Table 1.

### Toxins and ribotypes diversity

**Figure 1:**
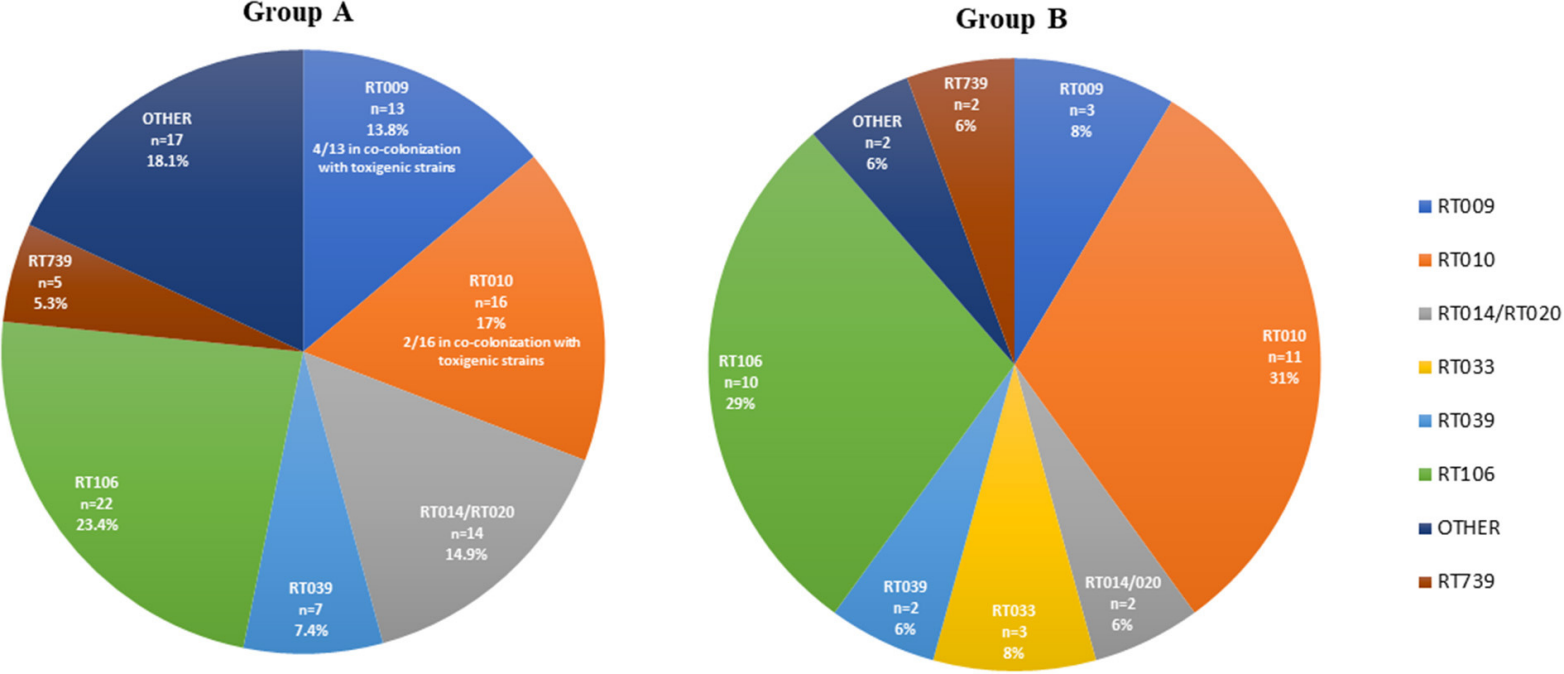
Ribotype diversity of *Clostridioides difficile* strains isolated from companion animals in group A (veterinary hospitals, *n* = 94) and group B (veterinary diagnostic laboratory, *n* = 35). Other = all other ribotypes with prevalence < 5%.

For group A, the toxins profile revealed that 50% (38/76) of the animals which were positive for *C. difficile* harbored strains carrying the toxins A and B genes (*tcdA*+*, tcdB*+), 38.2% (29/76) harbored non-toxigenic (*tcdA-, tcdB-*) strains and 11.8% (9/76) were carriers of both types of strains. No strain was positive for the binary toxin genes *cdtA* and *cdtB*. From the 76 positive samples of group A, a total of 94 isolates were obtained. The distribution of RTs is depicted in Figure 1, the most frequently detected toxigenic RTs were RT106 and RT014, and the non-toxigenic RT010 and RT009. Mixed carriage with toxigenic and non-toxigenic RTs was the most commonly found combination (52.9%, 9/17). Particularizing by animal species, the positive felines had a higher toxigenic carriage rate (66.7%, 8/12) than positive dogs (46.9%, 30/64). In both animal species the most commonly found RT was RT106.

Concerning group B, 52.9% (18/34) of the positive animals were carriers of toxigenic (*tcdA*+*, tcdB*+ and or *cdtA/B*+) *C. difficile* strains, while non-toxigenic strains were found in 47.1% (16/34) of the animals. Co-carriage was only found in one animal, totalling 35 isolates from 34 positive animals. The most representative RTs were the toxigenic RT106, RT033 and RT014/020, and the non-toxigenic RT010 and RT009 (Figure 1). The toxigenic profile of the strains isolated from the 23 positive dogs of this group did not show any specific trend, considering that 52% (12/23) harbored non-toxigenic *C. difficile* strains and 48% (11/23) toxigenic strains. Following the same trend observed in group A, felines showed a considerably higher toxigenic carriage rate than canines, with toxigenic strains present in 64% (7/11) of cats. Regarding RT distribution, while in dogs the most frequent RT was RT010 (39%, 9/23), followed by RT106 (22%, 5/23), in cats RT106 accounted for 45% of the cases and RT010 only for 18% (2/11).

Overall, considering the sum of the isolates from the two groups (*n* = 129), the two toxigenic RTs most commonly found were RT106 (24.8%) and RT014/020 (11.6%).

### Antimicrobial susceptibility and genetic determinants of resistance

In group A (Table 2), the highest rate of resistance was observed for clindamycin, 25.5% (24/94), moxifloxacin and metronidazole resistance followed, with 13.8% (13/94) and 12.8% (12/94), respectively. Only 2.1% (2/94) of the isolates were resistant to rifampicin. Few isolates revealed resistance to more than one of the antibiotics tested, with 10.6% (10/94) being resistant to both metronidazole and clindamycin, 1.1% (1/94) to moxifloxacin and clindamycin and 3.2% (3/94) being simultaneously resistant to three antibiotics (multidrug resistant, MDR). Resistance to metronidazole and clindamycin was mainly found in RT010, while moxifloxacin resistance was predominantly found in RT106.

**Table 2:** Antimicrobial resistance prevalence and genetic determinants of resistance in groups A and B.

**Resistant phenotype**	**Group A % (n/N)**	**Group B % (n/N)**	**Total % (n/N)**	AMR genetic determinants ^*^(n isolates)	**Main RT associated (n isolates)**
Clindamycin	25.5% (24/94)	34.3% (12/35)	27.9% (36/129)	*ermB* (36)	RT010 (23)
Moxifloxacin	13.8% (13/94)	8.6% (3/35)	12.4% (16/129)	*gyrA* Thr82Ile (16)	RT106 (8)
Metronidazole	12.8% (12/94)	28.6% (10/35)	17.1% (22/129)	pCD-METRO plasmid (22)	RT010 (22)
Rifampicin	2.1% (2/94)	-	2.1% (2/94)	*rpoB* Arg505Lys (1) *rpoB* His502Asn and Arg505Lys (1)	N.A.

^*^Determined by PCR and/or Sanger sequencing. The *gyrA* mutation in RT106 isolates was confirmed by WGS.

N.A. there was no main associated RT.

In group B (Table 2), the highest resistance rate was detected for clindamycin, 34.3% (12/35), followed by metronidazole, 28.6% (10/35). Resistance to moxifloxacin was found in 8.6% (3/35) of the *C. difficile* isolates. Regarding combined resistance, 20% (7/35) of the isolates were resistant to metronidazole and clindamycin and 2.9% (1/35) were resistant to moxifloxacin and clindamycin. A total of 5.7% (2/35) of the isolates were MDR. Similarly to group A, resistance to metronidazole was exclusively found in RT010 isolates, while clindamycin resistance showed a wider distribution among RTs but were still mainly represented by RT010 isolates.

Concerning AMR determinants in companion animal's isolates (Table 2), resistance to clindamycin was associated with the presence of the *ermB* gene, and resistance to moxifloxacin and rifampicin were associated with known point mutations in *gyrA* and *rpoB*, respectively. All metronidazole resistant isolates from RT010 were confirmed to harbor the pCD-METRO plasmid. For one RT14/020 human isolate, the *in silico* analysis additionally identified the presence of *tetM*, conferring putative resistance to tetracycline. Moreover, most of the RT014/020 human and animal isolates (34/41) were found to display the C656T mutation on the *23S rRNA* gene, but no association was found with clindamycin resistance.

### *Clostridioides difficile* infection, ribotypes prevalence and trends in humans

In order to understand the epidemiological trend of common community circulating RTs holding zoonotic potential, including RT106 and RT014/020, we analyzed data from the Portuguese CDI surveillance network, comprising data from eight acute care hospitals from three areas of mainland Portugal (North, Center, Metropolitan Area of Lisbon), over an 8-year period (2014–2021). A total of 1,498 non-duplicate isolates were included in this analysis, from which 221 distinct RTs were identified (Supplementary Figure S1).

**Figure 2:**
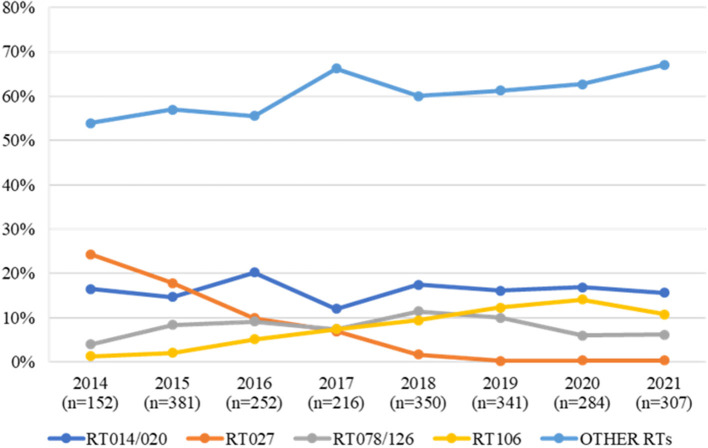
Trend of RT014/020, RT027, RT078/126 and RT106 strains isolated from human clinical infections over an 8-year period (2014–2021) in Portugal. All other ribotypes are compiled in the “OTHER RTs” category.

Considering the evolution of epidemic nosocomial strains vs. potentially zoonotic strains, we observed an inverse trend relative to the frequency of these types over the years (Figure 2). In fact, while the epidemic RT027 was the most prevalent RT in 2014 and 2015, accounting for 24.3% (37/152) and 17.9% (68/381) of all strains, from 2016 onwards a steady decline in RT027 prevalence has been observed, falling to residual prevalence values (< 0.5%). Contrary to this trend, when looking at RTs more associated with community acquisition, RT014/020 is one of the most commonly detected RTs in human infections in Portugal, with a considerable high prevalence over the years. Considering RT106, it has been steadily rising over the 8-year period considered, consistently assuming a place within the three most frequent RTs from 2017 onwards, accounting for 14.1% (40/279) of the isolates in 2020. It is also relevant to look at RT078/126 trend over the years as this is often regarded as one of the most important zoonotic RTs (6, 8, 9). This RT has remained relatively stable as one of the five most frequent RTs.

### Genetic diversity of *Clostridioides difficile* RT106 and RT014/020 isolates collected in Portugal

**Figure 3:**
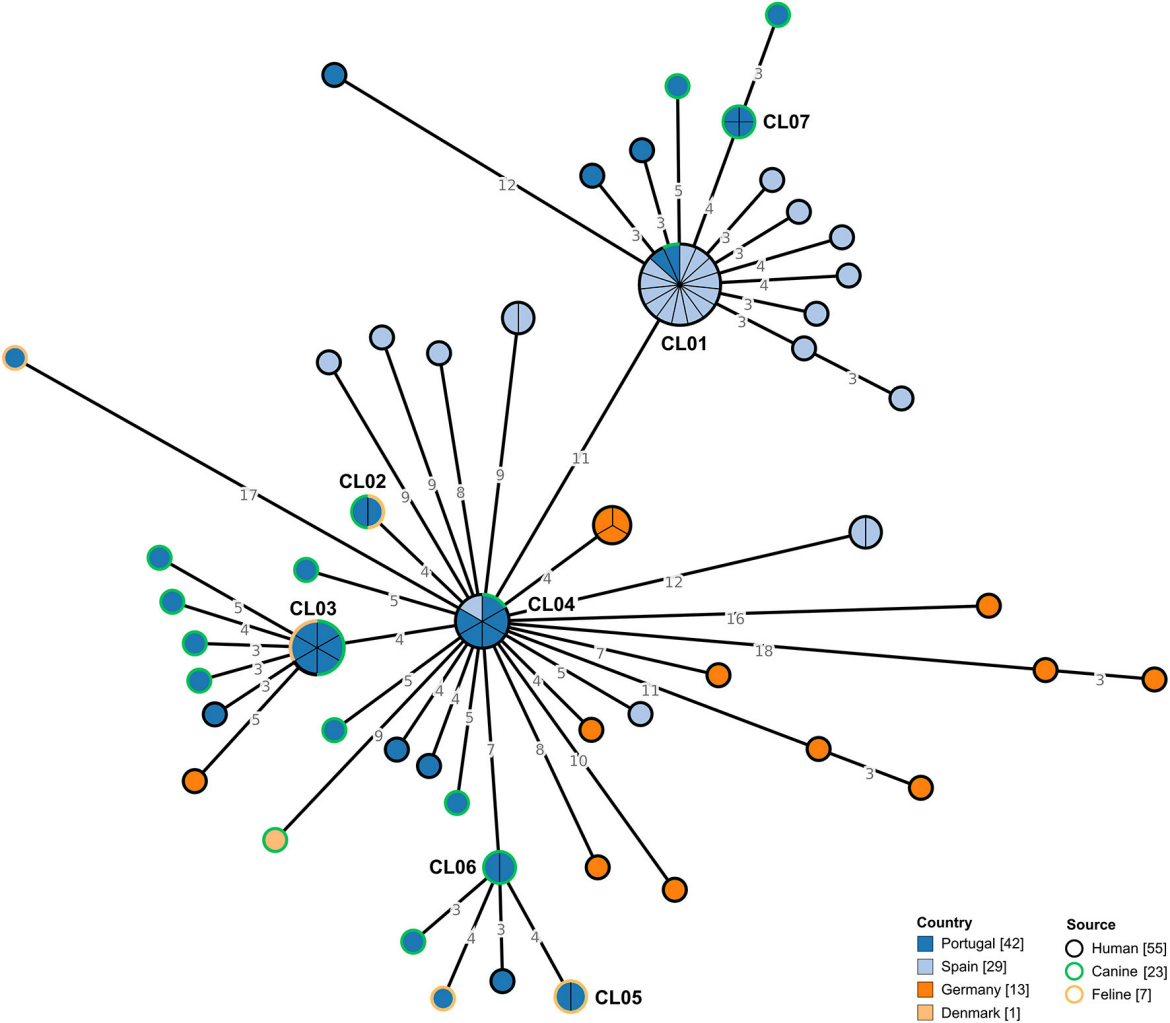
Phylogeny of *Clostridioides difficile* isolates from ribotype 106 used in the present study. The minimum spanning tree (MST) was constructed based on the core-SNP diversity found among 85 isolates, relative to reference genome DH/NAP11/106/ST-42 (CP022524.1). All nodes (which represent a unique allelic profile) presenting an SNP distance ≤ 2, representing clusters with potential epidemiological relevance, have been collapsed for visualization purposes. Nodes are colored according to different countries of origin and their contour colored by respective source. The MST was generated using GrapeTree v1.5.0 software (33).

Forty-two *C. difficile* RT106 isolates collected in Portugal, isolated from companion animals and humans, were subjected to WGS. The selected genomes were integrated with previously sequenced RT106 genomes (*n* = 43) from distinct countries (Figure 3). Data showed that circulating *C. difficile* isolates from Portugal were genetically diverse, being dispersed along the MST, with some isolates tightly clustering with isolates from Spain (CL01 and CL04). Moreover, we observed that isolates from distinct sources were also dispersed, consistent with a potential association between human and non-human isolates. In fact, when applying a ≤ 2 SNP threshold, seven closely related genetic clusters including isolates from Portugal could be observed (Figure 3, Table 3). Four of these clusters (CL01 to CL04), enrolled isolates from different sources, three of which included isolates from humans and companion animals (CL01, CL03, and CL04). Of note, cluster CL07 was composed by four distinct moxifloxacin resistant isolates collected from canines (Supplementary Table S1). Additionally, the largest observed cluster, CL01, enrolled not only isolates collected from different countries and sources, but also isolates spanning a 7-year time period (Table 3).

**Table 3:** Genetic clusters including *Clostridioides difficile* isolates from Portugal.

**Cluster ID**	**Cluster length (n°of isolates)**	**Samples**	**Ribo** **type**	**Country**	**Source**	**Collection date**	**Moxifloxacin resistance**
CL01	15	PT_CD00043, PT_CD00057, ERR3276441, ERR3276506, ERR3278163, ERR3278167, ERR3288184, ERR3288190, ERR3288338, ERR3289201, ERR3289202, ERR3289206, ERR3289207, ERR3289212, ERR3299518	106	Spain (86.7%), Portugal (13.3%)	Human (93.3%), Canine (6.7%)	2015 (46.7%), 2014 (33.3%), 2021 (13.3%), 2016 (6.7%)	No data (86.7%), R (13.3%)
CL02	2	PT_CD00053, PT_CD00055	106	Portugal (100.0%)	Feline (50.0%), Canine (50.0%)	2021 (100.0%)	S (100.0%)
CL03	6	PT_CD00064, PT_CD00030, PT_CD00031, PT_CD00048, PT_CD00049, PT_CD00044	106	Portugal (100.0%)	Canine (50.0%), Feline (33.3%), Human (16.7%)	2021 (66.7%), 2020 (33.3%)	S (83.3%), R (16.7%)
CL04	6	PT_CD00059, PT_CD00060, PT_CD00062, PT_CD00066, PT_CD00046, ERR3288329	106	Portugal (83.3%), Spain (16.7%)	Human (83.3%), Canine (16.7%)	2021 (83.3%), 2014 (16.7%)	S (66.7%), R (16.7%), No data (16.7%)
CL05	2	PT_CD00027, PT_CD00028	106	Portugal (100.0%)	Feline (100.0%)	2020 (100.0%)	S (100.0%)
CL06	2	PT_CD00036, PT_CD00029	106	Portugal (100.0%)	Canine (100.0%)	2021 (50.0%), 2020 (50.0%)	S (100.0%)
CL07	4	PT_CD00050, PT_CD00051, PT_CD00052, PT_CD00054	106	Portugal (100.0%)	Canine (100.0%)	2021 (100.0%)	R (100.0%)
CL08	2	PT_CD00094, PT_CD00095	014/020	Portugal (100.0%)	Feline (50.0%), Canine (50.0%)	2021 (100.0%)	S (100.0%)
CL09	2	PT_CD00086, PT_CD00098	014/020	Portugal (100.0%)	Canine (50.0%), Human (50.0%)	2021 (100.0%)	R (50.0%), S (50.0%)
CL10	3	PT_CD00075, PT_CD00077, PT_CD00085	014/020	Portugal (100.0%)	Human (100.0%)	2021 (100.0%)	S (100.0%)
CL11	2	PT_CD00099, PT_CD00100	014/020	Portugal (100.0%)	Human (100.0%)	2021 (100.0%)	S (100.0%)
CL12	6	PT_CD00089, PT_CD00091, PT_CD00093, ERR1307028, SRR7308801, SRR7309218	014/020	Portugal (50.0%), Australia (16.7%), Ireland (16.7%), Italy (16.7%)	Canine (50.0%), Human (50.0%)	2021 (50.0%), 2013 (33.3%), 2012 (16.7%)	No data (50.0%), S (33.3%), R (16.7%)
CL13	2	PT_CD00108, PT_CD00107	014/020	Portugal (100.0%)	Feline (50.0%), Canine (50.0%)	2021 (100.0%)	S (100.0%)
CL14	2	PT_CD00088, ERR3465438	014/020	Portugal (50.0%), Germany (50.0%)	Canine (50.0%), Human (50.0%)	2021 (50.0%), 2014 (50.0%)	S (50.0%), No data (50.0%)
CL15	3	PT_CD00071, PT_CD00073, PT_CD00074	014/020	Portugal (100.0%)	Human (100.0%)	2021 (100.0%)	S (100.0%)

R, Resistant; S, Susceptible. Genetic clusters were defined at a threshold of ≤ 2 SNPs, as previously proposed (29).

All isolates from Portugal were collected in 2021, except samples from two felines from CL03 that were collected in 2020.

**Figure 4:**
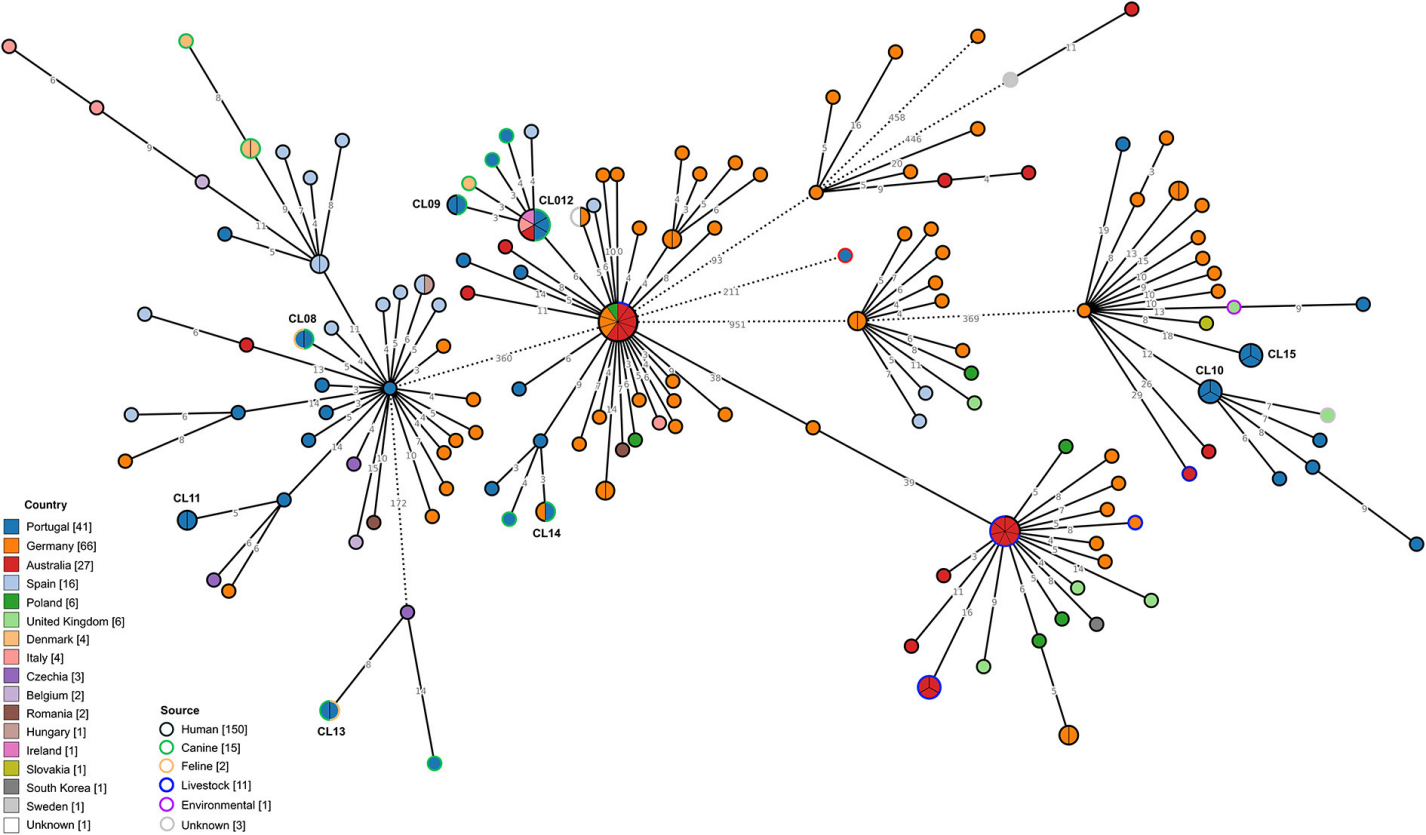
Phylogeny of *Clostridioides difficile* isolates from ribotypes 014/020 used in the present study. The minimum spanning tree (MST) was constructed based on the core-SNP diversity found among 183 isolates, relative to reference genome S-0352 (CP076377.1). All nodes (which represent a unique allelic profile) presenting an SNP distance ≤ 2, representing clusters with potential epidemiological relevance, have been collapsed for visualization purposes. Straight and dotted lines reflect nodes linked with the SNP distances below and above 100 respectively. Nodes are colored according to different countries of origin and their contour colored by respective source. The MST was generated using GrapeTree v1.5.0 software (33).

Regarding *C. difficile* RT014/020, 41 genomes from isolates collected from distinct sources in Portugal were integrated with 142 genomes previously obtained in several countries (Figure 4). Similarly to what was observed for RT106 isolates, RT014/020 isolates from Portugal presented considerable genetic diversity. Clustering data at a 2 SNP threshold revealed eight distinct genetic clusters enrolling isolates from Portugal, two of which also comprised isolates from other countries (Figure 4, Table 3), namely Germany, Australia, Italy and Ireland. Three out of the five clusters enrolling isolates from distinct sources linked genomes from companion animals and human infection cases (CL09, CL12, and CL14).

Overall, for the 14 genetic clusters including at least two isolates from Portugal, there was overlapping of Portuguese geographical regions, except for three cases (CL03, CL04, and CL09) (Supplementary Table S1).

Regarding mobile genetic elements (MGEs), no plasmids were identified, while phages were found for both RT106 and RT014/020, but no association could be established with the RT or the source of the isolates (data not shown). Moreover, as expected for RT106, all isolates carried the characteristic GI1 but none possessed the GI2 (Supplementary Table S1). Regarding GI3, it was present in 40.5% (17/42) of the isolates, but again no association was found with the origin of the isolate.

## Discussion

This study aimed to evaluate the frequency of *C. difficile* in companion animals, characterizing the circulating strains, and assessing the genetic overlap between animal and human strains in order to understand the role of companion animals in the community associated transmission network and the potential public health implications. The results from groups A and B reveal a positivity rate of 26% (76/292) and 18.6% (34/183), respectively, though around 40% of these animals only carried non-toxigenic strains. This is in accordance with previous studies reporting that non-toxigenic RTs, like RT009 and RT010, account for a considerable portion of companion animal associated *C. difficile* strains (16, 45, 46). The positivity rate noted in the population considered in this study contrasts with most previous findings often reporting *C. difficile* rates below 10% in companion animal (17, 47–49). Even though there are sporadic reports of significantly higher positivity rates (50) these usually apply to specific animal populations subjected to environmental conditions that may influence *C. difficile* isolation rate, thus being less representative of the general animal population. Although the convenience sampling methodology applied in this study can be considered a limitation by not allowing for a real prevalence estimate, it is in line with the sampling strategy applied in the studies abovementioned, allowing comparison of results.

Regarding host species specific tendencies, our results support previous studies in which no species predisposition was noted (16, 48, 51). Studies including felines are sparse and most commonly report low isolation rates (≤ 10%) (52). Our results (21.2% (12/57) in group A; 13.3% (11/83) in group B) show that a considerable percentage of cats can carry this pathogen, which should be taken into account when considering litter box sanitation practices. In addition, in our study, the isolation of toxigenic strains from cats was considerably higher than those isolated from dogs, meaning that feline feces may hold a higher pathogenic potential if interspecies *C. difficile* transmission occurs. Regarding the presence of gastrointestinal signs of disease, there was no significant association between the presence of diarrhea or altered fecal consistency and the isolation of *C. difficile*. These results corroborate the asymptomatic carrier status documented by previous authors (53, 54) and reinforces the need of raising awareness to the potential risks associated with companion animal's waste disposal, especially when it comes to seemingly normal fecal material that is inevitably accompanied by a lower hazard perception by the general public. The genotyping analysis noting a high prevalence of toxigenic strains, 50% (38/76) in group A and 52.9% (18/34) in group B, raises an alert about the pathogenic potential of animal associated strains.

Analyzing the genetic diversity at RT level within each group, it is possible to note a clear tendency, as the same RTs were predominant in both groups. The RT diversity reported among companion animals varies between studies but RT106 and RT014/020 are regularly identified (17, 53). These two RTs were amongst the most representative RTs in both groups, with RT106 being the most frequently detected in group A (Figure 1). Apart from the virulence associated with toxin production, RT106 was the most representative RT concerning moxifloxacin resistance, which may constitute an additional public health concern. Recent reports indicating an increasing prevalence of these RTs in human CA-CDI (55, 56) have gathered international attention. Data from the Portuguese surveillance programme supports a changing *C. difficile* epidemiology with epidemic RTs steadily decreasing over time and giving way to RTs with more zoonotic potential, like RT014/020 and RT106 (Figure 2). Considering that the RTs circulating in the studied companion animal's population are the same the ones most commonly isolated in humans, it is of paramount importance to understand the possible link between them and to assess the possibility and direction of interspecies transmission.

The RT033 strains isolated in group B also deems some attention as these, contrasting to the classical RT033, were positive for both *tcdA* and *tcdB*. To our knowledge this is only the second time a toxigenic RT033 strain has been described in animals, following its recent report in a swine production unit (57). The apparent wider tropism of this toxigenic strain for different animal hosts deserves further investigations in order to understand the genetic relatedness between these strains and the role they assume in the animal transmission chain.

Non-toxigenic RT009 and RT010 were also amongst the most commonly isolated RTs in both groups, corroborating previous studies (46). Even though the lack of toxin production implies a reduced public health risk, it is important to note the high rate of metronidazole and clindamycin resistance found in RT010 isolates. Metronidazole resistant RT010 isolates have been previously reported in canines (49, 58) and may assume a particularly important role in CDI epidemiology. Although this drug is no longer recommended as the first treatment option for CDI due to high associated recurrence rates, it is still the preferred choice if fidaxomicin and vancomycin are not available (59). Furthermore, the mobile nature of the associated AMR genetic determinants (pCD-METRO plasmid and *ermB* gene) may pose the additional risk of horizontal transmission between resistant and susceptible strains (41). These transmission events may be present in the gastrointestinal environment of animals carrying both toxigenic and non-toxigenic strains, which accounted for 12.5% (8/64) of the positive canines from group A.

Regarding fluoroquinolone resistance, GyrA Thr82Ile was present in all the moxifloxacin resistant strains, with the exception of a human isolate harboring the more rarely reported GyrA Asp81Asn mutation (60). The high rates of moxifloxacin resistance observed in both animal groups contrast with most studies focused on companion animal populations, which tend to report an overall susceptibility to this antibiotic (14, 61). The lack of accurate and official data on the use of fluoroquinolone in companion animals in Portugal makes it hard to correlate these results with antimicrobial use practices.

The results from the WGS-based analysis revealed that strains belonging to the same RT are genetically diverse, demonstrating that no assumption regarding genetic proximity should be made at RT level. A detailed genomic analysis at SNP level reveals that the strains collected in Portugal are dispersed widely within the MST, with no clear tendency to cluster based on source (Figures 3, 4). Clustering analysis suggests that the interspecies clonal transmission (≤ 2 SNP) of *C. difficile* strains between companion animals and humans in either direction is possible, with several identified clusters harboring isolates from distinct sources (Table 3). Interestingly, two of the clusters including strains from Portugal (CL01 and CL04) also included human strains from Spain. CL01 is the biggest cluster observed in the RT106 MST and it covers isolates from a 7-year period, suggesting that the RT106 may be well established within this community. These could also argue in favor of a common environmental contamination source as both countries share a big portion of natural resources and wildlife habitats. The fact that most moxifloxacin resistant isolates from companion animals belong to RT106 deserves further investigation, and may constitute an additional public health concern to the community circulation of these strains, as fluoroquinolone resistant strains have been associated with nosocomial outbreaks (62).

Regarding RT106 MGEs, all sequenced strains harbored the recently described RT specific GI1. Considering that the genes encoded within this GI may influence bacterial behavior concerning AMR and biofilm formation, it is possible that the presence of this MGEs grants advantages related to intestinal colonization and environmental fitness, contributing to the establishment of a robust community associated transmission network. Population characteristics might have accounted for the absence of GI2 in all sequenced isolates, considering it is mainly present in pediatric human isolates (44) which are underrepresented in this study. The fact that no association could be established between the presence of GI1, GI2 and GI3 and the host species reinforces the idea of a dynamic interspecies bacterial community with an evolutionary path that was not influenced by host specificity.

Considering the RT014/020 MST, a much wider distribution of the isolates from Portugal can be found (Figure 4). The observed genetic diversity could be a consequence of the broader range of hosts reported for this RT (63, 64), each contributing to the expansion of the transmission network. Clustering at 2 SNP level revealed eight different clonal clusters containing strains from Portugal, with CL09, CL012, and CL014 including human and canine strains. Even though these results could suggest the possibility of interspecies transmission between companion animals and humans, the absence of geographic link between isolates does not support such events for RT014. Nevertheless, previous reports on the genetic proximity between human and animal RT014/020 *C. difficile* strains had already suggested the possibility of animal to human transmission based on allelic differences (14). The existence of additional international clusters also comprising strains from animal and human origin, indicates that the interspecies transmission of *C. difficile* may not be an isolated event but a possible transmission route that can be established if circumstances allow. The fact that the Portuguese RT014/020 strains cluster with strains from a much higher variety of countries in comparison to RT106, supports the idea that RT014/020 is much better established internationally.

It is important to mention that for these clusters to constitute clear evidence of interspecies transmission an epidemiological link between the hosts would have to be asserted, which was not possible in this study. The role of a common environmental source of contamination cannot be excluded, especially if we consider the intimate connection held between companion animals and their owners at household level. Even though clear evidence of the zoonotic potential of *C. difficile* are lacking, previous studies suggest that interspecies transmission would have to be established in both directions (human to animal and animal to human) (65–68), supporting the idea that animal owning domestic environments might be associated with transmission events. Also, as shown by previous environmentally focused studies, the role of dogs as a vehicle of *C. difficile* spores dissemination in public spaces should not be overlooked (69).

## Conclusion

The present study represents an important contribution to the overall knowledge on the epidemiological role of companion animals in CA-CDI and brings awareness to the importance of including companion animals in the One Health research. With the number of animals owning households expected to increase in the coming years it is of paramount importance to clarify their role in community pathogen transmission networks. The limitations of the present study should be however addressed by others wishing to build on this topic. These included the convenience sampling methodology, the lack of randomization in the selection of the individuals and animals, the inclusion of a single sample per animal making it impossible to determine the precise status of the positive animals and its implications for public health, as well as the inclusion of animal and human samples without a known epidemiological link. Due to these limitations, it was not possible to establish a definite transmission route between companion animals and humans, but the data here presented is highly suggestive of such possibility and require further investigation, while also assessing efficient and reasonable public health measures to minimize the risks associated with companion animals' waste disposal and advise on hygienic measures toward a safer animal-human interaction.

## Data availability statement

The datasets presented in this study can be found in online repositories. The names of the repository/repositories and accession number(s) can be found in the article/Supplementary material.

## Ethics statement

Ethical review and approval was not required for the animal study because the current study uses surplus fecal material of dogs and cats that were subjected to sampling for diagnostic purposes as prescribed by a veterinary and according to their clinical conditions. No additional sample was taken for the purpose of the study. In addition, the use of this material follows the practices of the veterinary hospitals involved in the study, which includes that, before sampling, owners must provide an informed consent agreeing with the use of surplus of fecal samples for investigation purposes. Written informed consent for participation was not obtained from the owners because the written consent was obtained for the use of the surplus fecal samples for investigation purposes, but not only in the context of this study.

## Author contributions

FA, RC, and MOle conceptualized the study. FA, MP, AN, and MOle worked on the original draft of the manuscript. Reviewed and edited by FA, MP, AN, CP, MOli, LS, JG, and MOle. All authors contributed to the methodology. All authors have read and agreed to the published version of the manuscript.

## Supplementary material

Supplementary Figure S1 Percentage prevalence by year of *Clostridioides difficile* ribotypes from human clinical infections over an 8-year period (2014–2021) in Portugal. Other = all other ribotypes with prevalence < 3%.

## References

[1] Lance George W Goldstein EJC Sutter VL Ludwig SL Finegold SM. Ætiology of antimicrobial-agent-associated colitis. Lancet. (1978) 311:802–3. 10.1016/S0140-6736(78)93001-5 85818

[2] Voth DE Ballard JD. Clostridium difficile toxins: mechanism of action and role in disease. Clin Microbiol Rev. (2005) 18:247–63. 10.1128/CMR.18.2.247-263.2005 15831824 PMC1082799

[3] Buffie CG Jarchum I Equinda M Lipuma L Gobourne A Viale A. Profound alterations of intestinal microbiota following a single dose of clindamycin results in sustained susceptibility to clostridium difficile-induced colitis. Infect Immun. (2012) 80:62–73. 10.1128/IAI.05496-11 22006564 PMC3255689

[4] Ofori E Ramai D Dhawan M Mustafa F Gasperino J Reddy M. Community-acquired Clostridium difficile : epidemiology, ribotype, risk factors, hospital and intensive care unit outcomes, and current and emerging therapies. J Hosp Infect. (2018) 99:436–42 10.1016/j.jhin.2018.01.015 29410012

[5] Khanna S Pardi DS Aronson SL Kammer PP Orenstein R St Sauver JL. The epidemiology of community-acquired clostridium difficile infection: a population-based study. Am J Gastroenterol. (2012) 107:89–95. 10.1038/ajg.2011.398 22108454 PMC3273904

[6] Koene MGJ Mevius D Wagenaar JA Harmanus C Hensgens MPM Meetsma AM. Clostridium difficile in Dutch animals: their presence, characteristics and similarities with human isolates. Clin Microbiol Infect. (2012) 18:778–84. 10.1111/j.1469-0691.2011.03651.x 21919997

[7] Schneeberg A Neubauer H Schmoock G Baier S Harlizius J Nienhoff H. Clostridium difficile genotypes in piglet populations in Germany Alexander. J Clin Microbiol. (2013) 51:3796–803. 10.1128/JCM.01440-13 24025903 PMC3889769

[8] Rodriguez C Taminiau B Van Broeck J Avesani V Delmée M Daube G. Clostridium difficile in young farm animals and slaughter animals in Belgium. Anaerobe. (2012) 18:621–5. 10.1016/j.anaerobe.2012.09.008 23041559

[9] Rodriguez C Avesani V Van Broeck J Taminiau B Delmée M Daube G. Presence of Clostridium difficile in pigs and cattle intestinal contents and carcass contamination at the slaughterhouse in Belgium. Int J Food Microbiol. (2013) 166:256–62. 10.1016/j.ijfoodmicro.2013.07.017 23973837

[10] Rodriguez-Palacios A Mo KQ Shah BU Msuya J Bijedic N Deshpande A. Global and Historical Distribution of Clostridioides difficile in the Human Diet (1981–2019): systematic review and meta-analysis of 21886 samples reveal sources of heterogeneity, high-risk foods, and unexpected higher prevalence toward the tropic. Front Med. (2020) 7:9 10.3389/fmed.2020.00009 32175321 PMC7056907

[11] Janezic S Potocnik M Zidaric V Rupnik M. Highly divergent clostridium difficile strains isolated from the environment. PLoS ONE. (2016) 11:1–12. 10.1371/journal.pone.0167101 27880843 PMC5120845

[12] Candel-Pérez C Ros-Berruezo G Martínez-Graciá C. A review of Clostridioides [Clostridium] difficile occurrence through the food chain. Food Microbiol. (2019) 77:118–29. 10.1016/j.fm.2018.08.012 30297042

[13] Weese JS Finley R Reid-Smith RR Janecko N Rousseau J. Evaluation of clostridium difficile in dogs and the household environment. Epidemiol Infect. (2010) 138:1100–4. 10.1017/S0950268809991312 19951453

[14] Bjöersdorff OG Lindberg S Kiil K Persson S Guardabassi L Damborg P. Dogs are carriers of Clostridioides difficile lineages associated with human community-acquired infections. Anaerobe. (2021) 67:317. 10.1016/j.anaerobe.2020.102317 33418077

[15] Marks SL Kather EJ Kass PH Melli AC. Genotypic and phenotypic characterization of clostridium perfringens and clostridium difficile in diarrheic and healthy dogs. J Vet Intern Med. (2002) 16:533–40. 10.1111/j.1939-1676.2002.tb02383.x 12322702

[16] Clooten J Kruth S Arroyo L Weese JS. Prevalence and risk factors for Clostridium difficile colonization in dogs and cats hospitalized in an intensive care unit. Vet Microbiol. (2008) 129:209–14. 10.1016/j.vetmic.2007.11.013 18164560

[17] Rabold D Espelage W Sin MA Eckmanns T Schneeberg A Neubauer H. The zoonotic potential of clostridium difficile from small companion animals and their owners. PLoS ONE. (2018) 13:1–12. 10.1371/journal.pone.0193411 29474439 PMC5825086

[18] Perrin J Buogo C Gallusser A Burnens AP Nicolet J. Intestinal carriage of clostridium difficile in neonate dogs. J Vet Med Ser B. (1993) 40:222–6. 10.1111/j.1439-0450.1993.tb00131.x 8342371

[19] Stone NE Sidak-Loftis LC Sahl JW Vazquez AJ Wiggins KB Gillece JD. More than 50% of clostridium difficile isolates from pet dogs in flagstaff, USA, carry toxigenic genotypes. PLoS ONE. (2016) 11:1–21. 10.1371/journal.pone.0164504 27723795 PMC5056695

[20] Persson S Torpdahl M Olsen KEP. Erratum: New multiplex PCR method for the detection of the Clostridium difficile toxin A (tcdA) and toxin B (tcdB) and the binary toxin (cdtA/cdtB) genes applied to a Danish strain collection. Clin Microbiol Infect. (2009) 15:296. 10.1111/j.1469-0691.2008.02092.x 19040478

[21] Bidet P Barbut F Lalande V Burghoffer B Petit JC. Development of a new PCR-ribotyping method for Clostridium difficile based on ribosomal RNA gene sequencing. FEMS Microbiol Lett. (1999) 175:261–6. 10.1111/j.1574-6968.1999.tb13629.x 10386377

[22] Fawley WN Knetsch CW MacCannell DR Harmanus C Du T Mulvey MR. Development and validation of an internationally-standardized, high-resolution capillary gel-based electrophoresis PCR-ribotyping protocol for Clostridium difficile. PLoS ONE. (2015) 10:1–14. 10.1371/journal.pone.0118150 25679978 PMC4332677

[23] Erikstrup LT Danielsen TKL Hall V Olsen KEP Kristensen B Kahlmeter G. Antimicrobial susceptibility testing of Clostridium difficile using EUCAST epidemiological cut-off values and disk diffusion correlates. Clin Microbiol Infect. (2012) 18:E266–72. 10.1111/j.1469-0691.2012.03907.x 22672504

[24] Hindler JA Schuetz AN Abbott A Antonara A Galas MF Rekasius VJ Humphries RM. Clinical Laboratory Standards Institute (CLSI) Subcommittee on Antimicrobial Susceptibility Testing. CLSI AST News Updat. (2016). Avialable online at: https://clsi.org/media/1700/clsi-news-winter-2016.pdf (accessed September 27, 2022).

[25] Zhou Z Alikhan NF Mohamed K Fan Y Achtman M. The EnteroBase user's guide, with case studies on Salmonella transmissions, Yersinia pestis phylogeny, and Escherichia core genomic diversity. Genome Res. (2020) 30:138–52. 10.1101/gr.251678.119 31809257 PMC6961584

[26] Frentrup M Zhou Z Steglich M Meier-Kolthoff JP Göker M Riedel T. A publicly accessible database for clostridioides difficile genome sequences supports tracing of transmission chains and epidemics. Microb Genomics. (2020) 6:1–13. 10.1099/mgen.0.000410 32726198 PMC7641423

[27] García-Fernández S Frentrup M Steglich M Gonzaga A Cobo M López-Fresneña N. Whole-genome sequencing reveals nosocomial Clostridioides difficile transmission and a previously unsuspected epidemic scenario. Sci Rep. (2019) 9:1–9. 10.1038/s41598-019-43464-4 31061423 PMC6502822

[28] Llarena A Ribeiro-Gonçalves BF Nuno Silva D Halkilahti J Machado MP Da Silva MS. INNUENDO: A cross-sectoral platform for the integration of genomics in the surveillance of food-borne pathogens. EFSA Support Publ. (2018) 15:1498. 10.2903/sp.efsa.2018.EN-1498

[29] Seemann T. Prokka: rapid prokaryotic genome annotation. Bioinformatics. (2014) 30:2068–9. 10.1093/bioinformatics/btu153 24642063

[30] Moloney G Eyre DW Aogáin MM McElroy MC Vaughan A Peto TEA. Human and porcine transmission of Clostridioides difficile Ribotype 078, Europe. Emerg Infect Dis. (2021) 27:2294–300. 10.3201/eid2709.203468 34423760 PMC8386809

[31] Bertelli C Laird MR Williams KP Lau BY Hoad G Winsor GL. IslandViewer 4: expanded prediction of genomic islands for larger-scale datasets. Nucleic Acids Res. (2017) 45:W30–5. 10.1093/nar/gkx343 28472413 PMC5570257

[32] Arndt D Grant JR Marcu A Sajed T Pon A Liang Y. A better, faster version of the PHAST phage search tool. Nucleic Acids Res. (2016) 44:W16–21. 10.1093/nar/gkw387 27141966 PMC4987931

[33] Zhou Z Alikhan NF Sergeant MJ Luhmann N Vaz C Francisco AP. Grapetree: Visualization of core genomic relationships among 100,000 bacterial pathogens. Genome Res. (2018) 28:1395–404. 10.1101/gr.232397.117 30049790 PMC6120633

[34] MixãoV Pinto M Gomes JP Borges V. ReporTree: a surveillance oriented tool to strengthen the linkage between pathogen genetic clusters and epidemiological data. Res Squ. [Preprint]. (2022). 10.21203/rs.3.rs-1404655/v1 PMC10273728 37322495

[35] Bortolaia V Kaas RS Ruppe E Roberts MC Schwarz S Cattoir V. ResFinder 40 for predictions of phenotypes from genotypes. J Antimicrob Chemother. (2020) 75:3491–500. 10.1093/jac/dkaa345 32780112 PMC7662176

[36] Doster E Lakin SM Dean CJ Wolfe C Young JG Boucher C. Morley PS. MEGARes 20: A database for classification of antimicrobial drug, biocide and metal resistance determinants in metagenomic sequence data. Nucleic Acids Res. (2020) 48:D561–9. 10.1093/nar/gkz1010 31722416 PMC7145535

[37] Gupta SK Padmanabhan BR Diene SM Lopez-Rojas R Kempf M Landraud L. ARG-annot, a new bioinformatic tool to discover antibiotic resistance genes in bacterial genomes. Antimicrob Agents Chemother. (2014) 58:212–20. 10.1128/AAC.01310-13 24145532 PMC3910750

[38] Alcock BP Raphenya AR Lau TTY Tsang KK Bouchard M Edalatmand A. CARD 2020: antibiotic resistome surveillance with the comprehensive antibiotic resistance database. Nucleic Acids Res. (2020) 48:D517–25. 10.1093/nar/gkz935 31665441 PMC7145624

[39] Curry SR Marsh JW Shutt KA Muto CA Mary M Leary O. High frequency of rifampin resistance identified in an epidemic clostridium difficile clone from a large teaching hospital. Clin Infect Dis. (2009) 48:315. 10.1086/596315 19140738 PMC2819169

[40] Spigaglia P Barbanti F Mastrantonio P Brazier JS Delme M Kuijper E. Fluoroquinolone resistance in Clostridium difficile isolates from a prospective study of C. difficile infections in Europe. J Med Microbiol. (2008) 57:784–9. 10.1099/jmm.0.47738-0 18480338

[41] Boekhoud IM Hornung BVH Sevilla E Harmanus C Bos-Sanders IMJG Terveer EM. Plasmid-mediated metronidazole resistance in Clostridioides difficile. Nat Commun. (2020) 11:1–12. 10.1038/s41467-020-14382-1 32001686 PMC6992631

[42] Carattoli A Zankari E Garciá-Fernández A Larsen MV Lund O Villa L. In Silico detection and typing of plasmids using plasmidfinder and plasmid multilocus sequence typing. Antimicrob Agents Chemother. (2014) 58:3895–903. 10.1128/AAC.02412-14 24777092 PMC4068535

[43] Roxas BAP Roxas JL Walker RC Harishankar A Mansoor A Anwar F. Phylogenomic analysis of Clostridioides difficile ribotype 106 strains reveals novel genetic islands and emergent phenotypes. Sci Rep. (2020) 10:1–17. 10.1038/s41598-020-79123-2 33335199 PMC7747571

[44] Kociolek LK Gerding DN Espinosa RO Patel SJ Shulman ST Ozer EA. Whole-genome analysis reveals the evolution and transmission of an MDR DH/NAP11/106 Clostridium difficile clone in a paediatric hospital. Clin Infect Dis. (2018) 73:1222–9. 10.1093/jac/dkx523 29342270 PMC6454525

[45] Keel K Brazier JS Post KW Weese S Songer JG. Prevalence of PCR ribotypes among clostridium difficile isolates from pigs, calves, and other species?. J Clin Microbiol. (2007) 45:1963–4. 10.1128/JCM.00224-07 17428945 PMC1933037

[46] Wetterwik KJ Trowald-Wigh G Fernström LL Krovacek K. Clostridium difficile in faeces from healthy dogs and dogs with diarrhea. Acta Vet Scand. (2013) 55:23. 10.1186/1751-0147-55-23 23497714 PMC3606456

[47] Andrés-Lasheras S Martín-Burriel I Mainar-Jaime RC Morales M Kuijper E Blanco JL. Preliminary studies on isolates of Clostridium difficile from dogs and exotic pets. BMC Vet Res. (2018) 14:1–8. 10.1186/s12917-018-1402-7 29523201 PMC5845233

[48] Álvarez-Pérez S Blanco JL Harmanus C Kuijper EJ García ME. Prevalence and characteristics of Clostridium perfringens and Clostridium difficile in dogs and cats attended in diverse veterinary clinics from the Madrid region. Anaerobe. (2017) 48:47–55. 10.1016/j.anaerobe.2017.06.023 28687280

[49] Orden C Blanco JL Álvarez-Pérez S Garcia ME Blanco JL Garcia-Sancho M. Isolation of Clostridium difficile from dogs with digestive disorders, including stable metronidazole-resistant strains. Anaerobe. (2017) 43:78–81. 10.1016/j.anaerobe.2016.12.008 27965048

[50] Mckenzie E Riehl J Banse H Kass PH Nelson S Marks SL. Prevalence of diarrhea and enteropathogens in racing sled dogs. J Vet Intern Med. (2010) 24:97–103. 10.1111/j.1939-1676.2009.0418.x 19925573

[51] Riley T V Adams JE O'neill GL Bowman RA. Gastrointestinal carriage of Clostridium difficile in cats and dogs attending veterinary clinics. Epidemiol Infect. (1991) 107:659–65. 10.1017/S0950268800049359 1752313 PMC2272098

[52] Madewell BR Bea JK Kraegel SA Winthrop M Tang YJ Silva J. Clostridium difficile : a survey of fecal carriage in cats in a veterinary medical teaching hospital. J Vet Diagnostic Investig. (1999) 54:50–4. 10.1177/104063879901100108 9925212

[53] Silva ROS Ribeiro MG de Paula CL Pires IH Oliveira Junior CA Diniz AN. Isolation of Clostridium perfringens and Clostridioides difficile in diarrheic and nondiarrheic cats. Anaerobe. (2020) 62:164. 10.1016/j.anaerobe.2020.102164 32151948

[54] Weese JS Staempfli HR Prescott JF Kruth SA Greenwood SJ Weese HE. The roles of Clostridium difficile and enterotoxigenic Clostridium perfringens in diarrhea in dogs. J Vet Intern Med. (2001) 15:374–8. 10.1111/j.1939-1676.2001.tb02332.x 11467596

[55] Davies KA Ashwin H Longshaw CM Burns DA Davis GL Wilcox MH. Diversity of clostridium difficile PCR ribotypes in europe: results from the European, multicentre, prospective, biannual, point-prevalence study of clostridium difficile infection in hospitalised patients with diarrhoea (EUCLID), 2012 and 2013. Eurosurveillance. (2016) 21:1–11. 10.2807/1560-7917.ES.2016.21.29.30294 27470194

[56] Carlson TJ Blasingame D. Gonzales-luna AJ, Alnezary F, Garey KW. Anaerobe Clostridioides dif fi cile ribotype 106 : A systematic review of the antimicrobial susceptibility, genetics, and clinical outcomes of this common worldwide strain. Anaerobe. (2020) 62:102142. 10.1016/j.anaerobe.2019.102142 32007682 PMC7153973

[57] Alves F Nunes A Castro R Sequeira A Moreira O Matias R. Assessment of the transmission dynamics of clostridioides difficile in a farm environment reveals the presence of a new toxigenic strain connected to swine production. Front Microbiol. (2022) 13:1–14. 10.3389/fmicb.2022.858310 35495679 PMC9050547

[58] Spigaglia P Drigo I Barbanti F Mastrantonio P Bano L Bacchin C. Antibiotic resistance patterns and PCR-ribotyping of Clostridium difficile strains isolated from swine and dogs in Italy. Anaerobe. (2015) 31:42–6. 10.1016/j.anaerobe.2014.10.003 25316022

[59] Johnson S Lavergne V Skinner AM Gonzales-luna AJ Garey KW Kelly CP Wilcox MH. Clinical Practice Guideline by the Infectious Diseases Society of America (IDSA) and Society for Healthcare Epidemiology of America (SHEA): 2021 Focused Update Guidelines on Management of Clostridioides difficile Infection in Adults. Clin Infect Dis. (2021) 73:e1029–441. 10.1093/cid/ciab549 34164674

[60] Vitro I Spigaglia P Barbanti F Louie T. Molecular analysis of the gyrA and gyrB quinolone resistance-determining regions of fluoroquinolone-resistant clostridium difficile mutants selected molecular analysis of the gyrA and gyrB quinolone resistance-determining regions of fluoroquinolone-resist. Antimicrob Agents Chemother. (2009) 53:2463–8. 10.1128/AAC.01252-08 19364867 PMC2687229

[61] Wei Y Sun M Zhang Y Gao J Kong F Liu D Yu H Du J. Prevalence, genotype and antimicrobial resistance of Clostridium difficile isolates from healthy pets in Eastern China. BMC Infect Dis. (2019) 10.1186/s12879-019-3678-z 30634930 PMC6330442

[62] Muto CA Pokrywka M Shutt K Mendelsohn AB Nouri K Posey K. A large outbreak of clostridium difficile-associated disease with an unexpected proportion of deaths and colectomies at a teaching hospital following increased fluoroquinolone use. Infect Cont Hosp Epidemiol. (2014) 26:273–80. 10.1086/502539 15796280

[63] Rodriguez Diaz C Seyboldt C Rupnik M. Non-human C. difficile reservoirs and sources: animals, food, environment. Adv Exp Med Biol. (2018) 1050:227–43. 10.1007/978-3-319-72799-8_13 29383672

[64] Knight DR Riley T V. Genomic delineation of zoonotic origins of Clostridium difficile. Front Public Heal. (2019) 7:1–16. 10.3389/fpubh.2019.00164 31281807 PMC6595230

[65] Loo VG Brassard P Miller MA. Household transmission of clostridium difficile to family members and domestic pets. Infect Control Hosp Epidemiol. (2016) 37: 10.1017/ice.2016.178 27767004

[66] Stoesser N Eyre DW Quan TP Godwin H Pill G Mbuvi E. Epidemiology of clostridium difficile in infants in Oxfordshire, UK : risk factors for colonization and carriage, and genetic overlap with regional C. difficile infection strains. PLoS ONE. (2017) 12:e0182307. 10.1371/journal.pone.0182307 28813461 PMC5559064

[67] Shaughnessy MK Bobr A Kuskowski MA Johnston BD Sadowsky MJ Khoruts A. Environmental contamination in households of patients with recurrent Clostridium difficile infection. Appl Environ Microbiol. (2016) 82:2686–92. 10.1128/AEM.03888-15 26921425 PMC4836413

[68] Rodríguez-Pallares S Fernández-Palacios P Jurado-Tarifa E Arroyo F Rodríguez-Iglesias MA Galán-Sánchez F. Transmission of toxigenic Clostridiodes difficile between a pet dog with diarrhea and a 10-month-old infant. Anaerobe. (2022) 74:19. 10.1016/j.anaerobe.2022.102519 35066151

[69] Orden C Neila C Blanco JL Álvarez-pérez S Harmanus C Kuijper EJ. Recreational sandboxes for children and dogs can be a source of epidemic ribotypes of Clostridium difficile. Zoonoses Public Health. (2017) 65:88–95. 10.1111/zph.12374 28686001

