# Supplementary figures and images for: Molecular epidemiology of Clostridioides difficile in companion animals: Genetic overlap with human strains and public health concerns
Source: Front Public Health. 2023 Jan 6;10:1070258. doi: 10.3389/fpubh.2022.1070258 (PMC9853383; doi:10.3389/fpubh.2022.1070258)

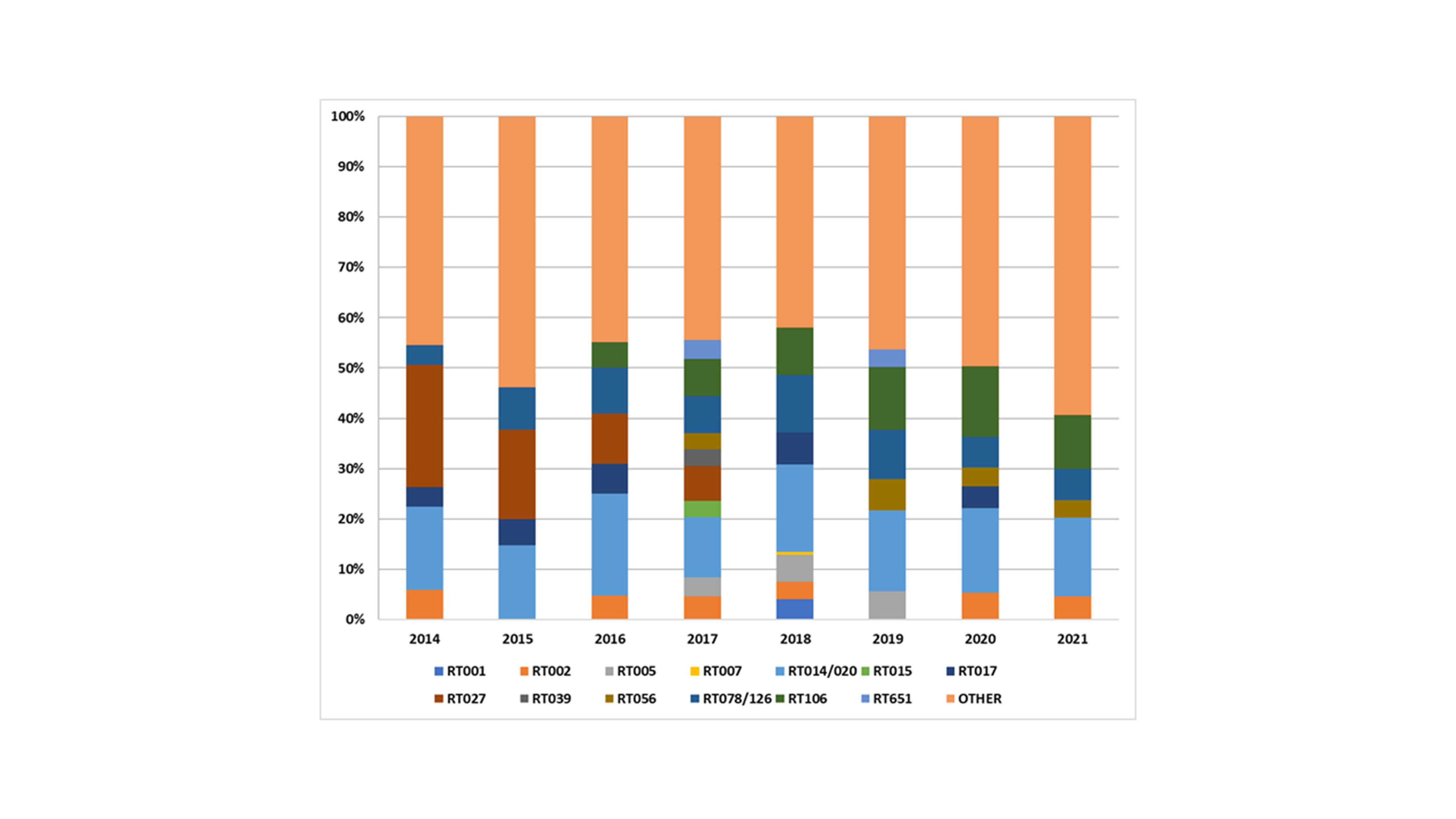

Supplement: Supplementary Figure S1 — Percentage prevalence by year of Clostridioides difficile ribotypes from human clinical infections over an 8-year period (2014–2021) in Portugal. Other = all other ribotypes with prevalence < 3%. [file Image_1.TIF]
